# Effects of the Antiobesity Drugs Aplex and Venera on Certain Biochemical and Physiological Indices in Obese Adult Male Albino Rats

**DOI:** 10.1155/2022/3776676

**Published:** 2022-04-11

**Authors:** Suzan S. A. Elpasty, Eman G. E. Helal, Ashraf M. M. Algendy, Hany N. Yousef

**Affiliations:** ^1^Department of Zoology, Faculty of Science (Girls), Al-Azhar University, Cairo, Egypt; ^2^Physiology Department, Faculty of Medicine (Boys), Al-Azhar University, Cairo, Egypt; ^3^Department of Biological and Geological Sciences, Faculty of Education, Ain Shams University, Cairo 11566, Egypt

## Abstract

**Background:**

Because of the growing incidence of obesity, the use of synthetic antiobesity medicines as weight-loss agents has grown in popularity, although their usefulness has yet to be established. Two of such medicines are Aplex and Venera. This study is designed to determine the potential dangers of Aplex and Venera on certain biochemical and physiological indicators in obese adult male rats.

**Methods:**

Twenty-one obese male albino rats (9 weeks old and having a body mass of 220 ± 20 g) were divided into three equal groups: the control group (vehicle treatment), the Aplex group (0.1 mg/kg/day) for 30 days, and the Venera group (0.1 mg/kg/day) for 30 days.

**Results:**

The values of serum glucose, insulin, homeostatic model assessment of insulin resistance (HOMA-IR), total protein, total cholesterol (TC), high-density lipoprotein (HDL), very-low-density lipoprotein (VLDL), TC/HDL ratio, testosterone, thyroxine (T4), and leptin did not differ significantly between the treated and control groups. In contrast, the treated groups had substantial changes in bodyweight, serum alanine aminotransaminase (ALT), aspartate aminotransaminase (AST), albumin, globulin, albumin/globulin ratio (A/G ratio), triglycerides (TG), low-density lipoproteins (LDL), LDL/HDL ratio, urea, creatinine, and triiodothyronine (T3) levels.

**Conclusion:**

The findings indicate that Aplex and Venera have negative impacts on crucial biochemical and physiological indicators, particularly liver and kidney functioning.

## 1. Introduction

Obesity is a major worldwide health issue that can affect both sexes during childhood, adolescence, and adulthood [1]. 600 million individuals worldwide are obese, while overweight adults account for 19 billion people [2]. A sedentary lifestyle and poor eating habits are the main causes of obesity leading to glucose intolerance, diabetes, hypertension, dyslipidemia, nonalcoholic fatty liver disease, coronary heart disease, and renal illnesses [3, 4].

Over the last few years, the use of antiobesity medicines to lower bodyweight by reducing food consumption or absorption or boosting energy expenditure has garnered attention [5]. The current antiobesity medicines have unfavorable side effects in high-risk cardiac patients, including pulmonary hypertension, valvular heart disease, psychological side effects, and an increased risk of heart attack and stroke [6].

Aplex is a popular slimming medication with an apple flavor. It comprises psyllium husk powder, apple juice, grapefruit juice, chromium, vitamin C, apple flavor, and sucralose. Psyllium husk, the main component of Aplex, has been utilized since prehistoric times throughout the world. Psyllium husk is the cleansed, dried seed coat separated from the seeds of Forska's Plantago ovate by winnowing and thrashing [7]. Psyllium, as a fiber, can help to avoid obesity and its consequences [8]. Sucralose, one of the most recent artificial sweeteners, is also present in Aplex [9].

Venera is another slimming medicine with a pineapple flavor and a dietary fiber supplement (psyllium husk) that is high in chromium and vitamin C. Chromium, the main component of Venera, is a trace mineral that has a function in carbohydrate, lipid, and protein metabolism [10].

The purpose of the current study is to see if the slimming medications Aplex and Venera have any detrimental impact on various biochemical and physiological markers that represent the metabolic condition of obese adult male albino rats.

## 2. Materials and Methods

### 2.1. Experimental Animals

Due to the variable nature of female data caused by hormonal fluctuations associated with the female estrous cycle, the current study was conducted on male rats only. Twenty-one male albino rats weighing 220 g with an average age of 9 weeks were used in this investigation. Rats were acquired from the Nile Pharmaceutical Company's animal house (Cairo, Egypt). They were kept in well-aerated polypropylene clear cages with seven rats in each cage, in a room with controlled conditions (a 12-hour light/dark cycle, a temperature range of 25 ± 2°C, and relative humidity of 55 ± 5%). For two weeks, they were provided with free access to a standard laboratory diet (54% carbohydrates, 22% proteins, 4.5% fat, 8% fibers, and 5% vitamins and minerals) and water before the commencement of the experiment, allowing them to adjust to the new habitat and detect any apparent signs of pathology.

All experimental procedures were performed in conformity with the principles and guidelines of the Ethics Committee of the Faculty of Science, Al-Azhar University, Cairo, Egypt, that conformed to the “Guide for the Care and Use of Laboratory Animals” published by the US National Institutes of Health (NIH publication no. 85–23, 1996) for the use and welfare of experimental animals.

### 2.2. The Applied Drugs

Aplex in the form of sachets (10 g/ sachet) was purchased from MedCare for Pharma Clinic Co. Registration number 6604/2016; while, Venera sachets (7 g/ sachet) were purchased from Pharma Zad, Industrial Area, Badr City, Cairo, for Advocure Pharmaceuticals.

### 2.3. Experimental Design

All rats utilized in this study were obese, with an average weight of 220 ± 20. They were separated into three equal groups of seven animals each, as follows:

  Group I (control group): throughout the experiment, each rat was given daily deionized distilled water (vehicle of Aplex and Venera) in the same manner as the treated groups
  Group II (Aplex group): each rat was treated orally with Aplex at a dosage of 0.1 mg/kg/12 hours for 30 days.
  Group III (Venera group): for 30 days, each rat was given Venera orally at a rate of 0.1 mg/kg/12 hours.

Based on normalization of dosage to the body surface area, an allometric scaling approach was applied to convert the pharmacological dose of Aplex and Venera from human to rat species [11].

### 2.4. Bodyweight (BW) Assessment

The animals were weighed each week of the experiment, and the initial and final assessments were determined. The following formula was used to calculate the percentage (%) change in bodyweight (BW):

(1) $$\begin{array}{c} \%\text{ change of the BW }=\frac{\text{final BW}-\text{initial BW}}{\text{initial BW}}\times 100. \end{array}$$

### 2.5. Collection of Sera

The animals were starved overnight at the end of the treatment period and afterwards anesthetized by light pentobarbitone anesthesia. Capillary tubes were used to obtain blood samples from the retroorbital venous plexus. The serum was separated by centrifuging the collected blood for 10 minutes at 5000 rpm, and the separated serum was aliquoted in an Eppendorf tube and kept frozen at −20°C until analysis.

### 2.6. Determination of Serum Biomarkers

#### 2.6.1. Pancreatic Function Biomarkers

Serum glucose concentrations were assayed following the glucose oxidase method [12]. Whereas, enzyme-linked immunosorbent assay (ELISA) kits (Abcam, catalog number: ab273188) were used to quantify serum insulin levels. The homeostatic model assessment of insulin resistance (HOMA-IR) was estimated using a free online calculator (HOMA Calculator, Version 2.2.3, Diabetes Trail Unit, The University of Oxford, Oxford, UK) depending on the following equation [13]:

(2) $$\begin{array}{c} \text{HOMA}-\text{IR}=\text{fasting serum insulin}\left(µ\text{IU}/\text{ml}\right)\times \frac{\text{fasting serum glucose}\left(\text{mmol}/\text{L}\right)\;}{22.5}. \end{array}$$

#### 2.6.2. Protein Profile

Using BioMerieux kits from France, total protein and albumin levels were assessed, and the concentration of serum globulin was calculated applying the following formula:

(3) $$\begin{array}{c} \text{Globulin }\left(\frac{\text{g}}{\text{dl}}\right)=\text{total protein }\left(\frac{\text{g}}{\text{dl}}\right)-\text{albumin }\left(\frac{\text{g}}{\text{dl}}\right). \end{array}$$

#### 2.6.3. Lipid Profile

Total cholesterol (TC), triglycerides (TG), and high-density lipoprotein (HDL) were measured according to the procedure describe by Allain et al. [14], Fossati and Prencipel [15], and Demacker et al. [16], respectively. Friedwald's et al. [17] and Norbert's [18] equations were used to calculate serum low-density lipoprotein (LDL) and very-low-density lipoprotein (VLDL). After calculating serum LDL and VLDL, the TC/HDL (risk factor 1) and LDL/HDL (risk factor 2) ratios were calculated.

(4) $$\begin{array}{c} \text{Friedewald's equation}:\text{LDL }\left(\frac{\text{mg}}{\text{dl}}\right)=\text{TC}-\left[\text{HDL}+\left(\frac{\text{TG}}{5}\right)\right], \end{array}$$

(5) $$\begin{array}{c} \text{Norbert equation}:\text{VLDL}=\frac{\text{TG}}{5}. \end{array}$$

#### 2.6.4. Liver Function Enzymes

Measuring the activities of aspartate aminotransaminase (AST) and alanine aminotransaminase (ALT) were based on protocols previously described by Tietz [19], using colorimetric assay kits (BioMerieux).

#### 2.6.5. Kidney Function Enzymes

Specific markers related to renal function including levels of urea and creatinine in the sera were assessed spectrophotometrically using commercial diagnostic kits (BioMerieux).

#### 2.6.6. Serum Testosterone, Triiodothyronine, Thyroxine, and Leptin

The level of serum testosterone was measured according to Vermeulen et al. [20], whereas serum triiodothyronine (T3) and thyroxine (T4) were estimated using ELISA Abcam kits as described previously [20]. Leptin level was estimated using a commercially available ELISA Kit (Crystal Chem Inc., USA).

### 2.7. Statistical Analysis

The biochemical data were expressed as mean ± SD (7 rats/group). The statistical variations between the control and test groups were evaluated by the independent samples *t*-test using IBM SPSS Statistics for Windows, version 22 (IBM Corp., Armonk, N.Y., USA).

## 3. Results

In terms of bodyweight, no significant difference in the percentage of bodyweight change was observed in animals treated with Aplex or Venera after 15 days, but a highly significant decrease (*P* < 0.01) in the percentage of bodyweight change was observed after 30 days when compared to control animals (Table 1).

Table 2 provides the serum glucose and insulin levels and the HOMA-IR values of the control and treated animal groups. In comparison with the control, Aplex or Venera administration had no significant (*P* > 0.05) impact on the assessed glycemic indices.

The data in Table 3 showed that the treatment of rats with Aplex or Venera induced a significant increase (*P* < 0.05) in the activity of AST and a highly significant increase (*P* < 0.01) in the activity of ALT in comparison with the control.

In the current study, serum protein profile characteristics (total protein, albumin, globulin, and A/G ratio) were estimated in control, Aplex, and Venera-treated rats, and the obtained results are given in Table 4. Animals given Aplex or Venera had no significant change in total protein but exhibited a significant decline (*P* < 0.05) in albumin and A/G ratio and a highly significant rise (*P* < 0.01) in globulin when compared with the control group.

Table 5 provides the lipid profile indices found in serum of rats from the control and experimental groups. In animals given Aplex or Venera, there was no significant change in total cholesterol, HDL, VLDL, or the TC/HDL ratio. In contrast, the current findings indicated a substantial drop (*P* < 0.05) in triglyceride levels in serum of rats given Aplex and a highly significant decrease (*P* < 0.01) in the case of the Venera group. LDL and LDL/HDL ratio decreased significantly (*P* < 0.05) in the Venera group compared with the control group.

Table 6 provides the serum urea and creatinine levels of the control and experimental groups. In comparison with the equivalent control rats, sera of Aplex-treated animals exhibited substantially (*P* < 0.05) higher amounts of urea and creatinine. In the case of Venera-treated animals, the reported rise in these renal markers became more evident in a highly significant manner (<0.01).

Table 7 provides the mean concentrations of testosterone, T3, T4, and leptin in sera of control and experimental animal groups. Aplex or Venera administration did not have significant (*P* > 0.05) effects on the levels of testosterone, T4, and leptin comparable with the control values. Meanwhile, T3 concentration was significantly elevated (*P* < 0.05) in Aplex and Venera groups compared with the values of control animals.

## 4. Discussion

Obesity is a cosmopolitan health problem caused by an imbalance between food intake and energy expenditure, resulting in exaggerated fat deposition in adipocytes, hepatocytes, muscles, pancreas, and other organs engaged in the metabolism, resulting in dyslipidemia, glucose intolerance, diabetes, hypertension, nonalcoholic fatty liver disease coronary heart disease, renal illnesses, and cancer [21, 22].

There are numerous antiobesity medications available; however, they have dangerous adverse effects [6]. The current study is designed to shed light on the potential negative effects of two slimming medications (Aplex and Venera) on the metabolic pattern of obese adult male albino rats through evaluating selected biochemical and physiological indicators in their sera.

Our findings show that there was a considerable decrease in bodyweight gain in rats administered Aplex or Venera in comparison with the control group. This may be attributed to the dietary fiber supplement psyllium (one of the major components of Aplex and Venera), which is a soluble fiber that suppresses appetite. These findings are consistent with Pal et al. [22] who discovered that Zucker rats fed a psyllium-supplemented diet for 25 weeks lost substantially more bodyweight than those fed the normal diet. Moreover, previous studies [23, 24] have reported that dietary fiber may influence weight gain by limiting energy intake, resulting in less weight gain. Furthermore, chromium, which is present in Aplex and Venera, may help to reduce bodyweight by increasing insulin sensitivity, promoting thermogenesis, and reducing appetite by activating insulin-sensitive glucoreceptors in the brain [25].

The current study's findings manifested that Aplex and Venera had no adverse effects on the measured pancreatic function biomarkers. In the same line, Hashem et al. [26] proved that the psyllium husk ethanolic extract has an ameliorative effect on the levels of glucose, insulin, HbA1c, and HOMA-IR in the sera of hyperlipidemic rats.

The liver is crucial in detoxification of endogenous waste products and exogenous substances [27]. Accordingly, because hepatocytes are the primary sites of the xenobiotic metabolism, they are particularly susceptible to toxicity caused by reactive metabolites. The current study revealed that both Aplex and Venera treatments induced a significant (*P* < 0.05) increase in the activity of AST and a highly significant (*P* < 0.01) increase in the activity of ALT relative to the corresponding control, which are the most suggestive indicators of hepatocyte structural degradation since these enzymes are found in the cytoplasm and are liberated into blood as a result of functional membrane integrity loss and cellular leaking [28]. Many substances, including clinically beneficial medicines, can induce hepatocellular damage by the metabolic activation of chemicals, resulting in highly reactive compounds such as free radicals, which produce oxidative stress [29]. In the current study, the elevated activities of AST and ALT may be attributed to the presence of chromium in Aplex and Venera which alters the activities of antioxidant enzymes and induces histological damage of the liver tissues resulting in biochemical disturbances in the levels of liver enzymes. Shafik et al. [30] discovered that chromium induces fat penetration in hepatocytes, resulting in hepatocellular injury. Furthermore, excess fat accumulation in hepatocytes may induce hepatic damage by direct cellular cytotoxicity, which is mediated by lipid peroxidation, free fatty acids, oxidative stress, and hepatotoxicity, resulting in liver dysfunction [31]. Our findings were consistent with previous studies [32, 33] which suggested that chromium might cause liver deterioration. Moreover, Aplex contains sucralose which is known as a hepatoxic agent in previous studies [9].

Treatment of rats with either Aplex or Venera caused disturbances in the protein profile pattern as reflected by the decline of albumin levels, A/G ratio, and elevation of globulin. This disturbance is a direct result of the liver tissue deterioration caused by the essential elements (sucralose, chromium, and psyllium) and (chromium and psyllium) found in Aplex and Venera, respectively. These findings are in line with those of other researchers [9, 30].

The present results revealed a significant decrease in TG in the sera of rats receiving Aplex and a highly significant decrease in the Venera group. Moreover, LDL and LDL/HDL ratio showed a significant decrease in the Venera group compared with the corresponding control group. The decrease in TG may be due to the presence of chromium in Aplex and Venera. The potentials of chromium to boost insulin sensitivity, reduce the hydrolysis of TG in adipocytes, and lower the amount of nonesterified fatty acids in blood are some of the hypotheses that have been proposed to explain the impact of chromium on lipids [34]. Furthermore, the reduced supply of nonesterified fatty acids to the liver reduces triglyceride and VLDL synthesis [34, 35]. In addition, the administration of Venera, which contains psyllium husk powder, causes a decrease in LDL levels, which agrees with Pal and Radavelli's findings [36]. Psyllium fiber has been shown in human and animal models to enhance dietary fiber consumption, which may lead to positive improvements in serum lipid and lipoprotein levels, including the mitigation of LDL. Additionally, Aplex contains sucralose that reduces the level of TG and LDL. The reduction in TG might be attributable to the sucralose action on peroxisome proliferator-activated receptors alpha (PPAR-*α*), which increases the production of lipoprotein lipase. Furthermore, PPAR-*γ* activation in adipose tissue promotes triglyceride accumulation [37].

Because the kidneys are engaged in purifying and concentrating many chemicals and substances that may reach high concentrations and become poisonous, they are extremely susceptible to the negative effects of chemicals and medicines [38]. The most common measures demonstrating proper kidney function are serum urea and creatinine levels [39]. As shown by the higher levels of the examined renal function indices, the findings of the current study revealed compromised kidney function in rats subjected to either Aplex or Venera compared with equivalent control rats. Chromium impairs renal function by lowering the glomerular filtration rate, which results in urea and creatinine retention in blood [40].

Amongst the evaluated hormones, T3 elevated markedly in the sera of rats subjected to either Aplex or Venera. Hyperthyroidism is caused by a high proportion of chromium in Aplex and Venera, which leads to harmful weight loss. These alterations in thyroid hormones might potentially be linked to a shift in the pituitary-thyroid axis as a result of chromium's stressful action [41, 42].

## 5. Conclusion

In conclusion, the findings of this study show that Aplex and Venera are capable of altering bodyweight and some crucial biochemical and physiological indicators, such as serum ALT, AST, albumin, globulin, A/G ratio, triglycerides, LDL, LDL/HDL ratio, urea, creatinine, and T3 levels.

## Figures and Tables

**Table 1:** Percentage bodyweight change in control and treated animal groups.

Parameters	Animal groups		
	Control (mean ± SD)	Aplex (mean ± SD)	Venera (mean ± SD)
Bodyweight change (15 days)	15.06 ± 2.38	18.98 ± 3.24	27.48 ± 5.06
% change		26.02%	82.47%

Bodyweight change (30 days)	62.40 ± 7.91	20.83 ± 3.89^*∗∗*^	24.45 ± 2.78^*∗∗*^
% change		−66.61%	−60.82%

Values are means ± SD of seven rats in each group. ^*∗*^*P* < 0.05, ^*∗∗*^*P* < 0.01 in comparison to the control group using the *t*-test.

**Table 2:** Pancreatic function biomarkers in sera of control and treated animal groups.

Parameters	Animal groups		
	Control (mean ± SD)	Aplex (mean ± SD)	Venera (mean ± SD)
Glucose (mg/dl)	102.60 ± 4.67	113.60 ± 7	115 ± 3.44
% change		10.72%	15.59%

Insulin (mu/ml)	0.93 ± 0.03	0.95 ± 0.03	0.95 ± 0.02
% change		1.93%	1.51%

HOMA-IR	0.24 ± 0.03	0.25 ± 0.01	0.26 ± 0.01
% change		4.10%	4.92%

Values are means ± SD of seven rats in each group. ^*∗*^*P* < 0.05, ^*∗∗*^*P* < 0.01 in comparison to the control group using the *t*-test. HOMA-IR, homeostatic model assessment of insulin resistance.

**Table 3:** Liver function enzymes in sera of control and treated animal groups.

Parameters	Animal groups		
	Control (mean ± SD)	Aplex (mean ± SD)	Venera (mean ± SD)
AST (U/L)	74.80 ± 10.76	79.40 ± 6.07^*∗*^	80.40 ± 7.73^*∗*^
% change		5.6%	7.48%

ALT (U/L)	27.20 ± 2.59	34.40 ± 3.21^*∗∗*^	35.60 ± 3.97^*∗∗*^
% change		26.47%	30.88%

Values are means ± SD of seven rats in each group. ^*∗*^*P* < 0.05, ^*∗∗*^*P* < 0.01 in comparison to the control group using the *t*-test. AST, aspartate transferase; ALT, alanine transferase.

**Table 4:** Protein profile indices in sera of control and treated animal groups.

Parameters	Animal groups		
	Control (mean ± SD)	Aplex (mean ± SD)	Venera (mean ± SD)
Total protein (g/dl)	6.38 ± 0.22	6.78 ± 0.48	6.74 ± 0.29
% change		6.27%	5.64%

Albumin (g/dl)	3.14 ± 0.15	2.80 ± 0.34^*∗*^	2.94 ± 0.27^*∗*^
% change		−10.83%	−6.37%

Globulin (g/dl)	3.24 ± 0.19	3.98 ± 0.22^*∗∗*^	3.80 ± 0.16^*∗∗*^
% change		22.84%	17.28%

A/G	0.97 ± 0.09	0.70 ± 0.07^*∗∗*^	0.78 ± 0.09^*∗∗*^
% change		−27.48%	−19.83%

Values are means ± SD of seven rats in each group. ^*∗*^*P* < 0.05, ^*∗∗*^*P* < 0.01 in comparison to the control group using the *t*-test. A/G, albumin/globulin ratio.

**Table 5:** Lipid profile indices in sera of control and treated animal groups.

Parameters	Animal groups		
	Control (mean ± SD)	Aplex (mean ± SD)	Venera (mean ± SD)
Total cholesterol (mg/dl)	86.40 ± 3.78	81.80 ± 3.96	86.80 ± 3.77
% change		−5.32%	−0.23%

TG (mg/dl)	135 ± 0.7	128 ± 0.9^*∗*^	120 ± 0.8^*∗∗*^
% change		−5%	−11%

HDL (mg/dl)	52.60 ± 6.58	48.20 ± 6.22	49.00 ± 4.47
% change		−8.37%	−6.84%

LDL (mg/dl)	13.64 ± 2.41	11.96 ± 2.67	7.44 ± 1.68^*∗*^
% change		−12.32%	−45.45%

VLDL (mg/dl)	27 ± 0.1	25.6 ± 0.8	24 ± 1.35
% change		−5%	−11%

TC/HDL	1.68 ± 0.20	1.72 ± 0.17	1.76 ± 0.13
% change		1.90%	4.51%

LDL/HDL	0.28 ± 0.03	0.26 ± 0.04	0.15 ± 0.02^*∗*^
% change		−7.14%	−46.48%

Values are means ± SD of seven rats in each group. ^*∗*^*P* < 0.05,^*∗∗*^*P* < 0.01 in comparison to the control group using the *t*-test. TG, triglycerides; HDL, high-density lipoproteins; LDL, low-density lipoproteins; VLDL, very-low-density lipoproteins.

**Table 6:** Kidney function enzymes in sera of control and treated animal groups.

Parameters	Animal groups		
	Control (mean ± SD)	Aplex (mean ± SD)	Venera (mean ± SD)
Urea (mg/dl)	37.3 ± 0.66	41.4 ± 1.1^*∗*^	53.4 ± 0.9^*∗∗*^
% change		11%	43%

Creatinine (mg/dl)	1.18 ± 0.025	1.5 ± 0.098^*∗*^	1.9 ± 0.02^*∗∗*^
% change		27%	62%

Values are means ± SD of seven rats in each group. ^*∗*^*P* < 0.05, ^*∗∗*^*P* < 0.01 in comparison to the control group using the *t*-test.

**Table 7:** Levels of testosterone, T3, T4, and leptin in sera of control and treated animal groups.

Parameters	Animal groups		
	Control (mean ± SD)	Aplex (mean ± SD)	Venera (mean ± SD)
Testosterone (ng/dl)	1.03 ± 0.08	1.06 ± 0.24	0.98 ± 0.031
% change		2.91%	−5%

T3 (ng/dl)	47.9 ± 0.6	55 ± 2.2^*∗*^	56 ± 2.4^*∗*^
% change		15%	19%

T4 (ng/dl)	3.02 ± 0.01	3.00 ± 0.59	3.16 ± 0.57
% change		−0.60%	4.71%

Leptin (ng/dl)	1.90 ± 0.35	2.10 ± 0.41	1.98 ± 0.38
% change		10.53%	4.21%

Values are means ± SD of seven rats in each group. ^*∗*^*P* < 0.05, ^*∗∗*^*P* < 0.01 in comparison to the control group using the *t*-test. T3, triiodothyronine; T4, thyroxine.

## Data Availability

The datasets used and/or analyzed during the current study are available from the corresponding author upon request.

## Additional Points

The research's limitation is that oxidative stress indicators were not examined; however, they will be measured and combined with other related parameters in a separate study that is now being conducted to continue searching at this point.

## References

[1] Guerrini Usubini A., Varallo G., Granese V. (2021). The impact of psychological flexibility on psychological well-being in adults with obesity. *Frontiers in Psychology*.

[2] Wang J.-B., Gu M.-J., Shen P. (2016). Body mass index and mortality: a 10-year prospective study in China. *Scientific Reports*.

[3] Romieu I., Dossus L., Barquera S. (2017). Energy balance and obesity: what are the main drivers?. *Cancer Causes Control*.

[4] Rosiek A., Maciejewska N., Leksowski K., Rosiek-Kryszewska A., Leksowski Ł. (2015). Effect of television on obesity and excess of weight and consequences of health. *International Journal of Environmental Research and Public Health*.

[5] Cooke D., Bloom S. (2006). The obesity pipeline: current strategies in the development of anti-obesity drugs. *Nature Reviews Drug Discovery*.

[6] Kang J. G., Park C.-Y. (2012). Anti-obesity drugs: a review about their effects and safety. *Diabetes & metabolism journal*.

[7] Madgulkar A. R., Rao M. R. P., Warrier D., Ramawat K. G., Mérillon J.-M. (2015). Characterization of psyllium (*Plantago ovata*) polysaccharide and its uses. *Polysaccharides*.

[8] Parwe S., Mohan M., Bhagwat P., Nisargandha M. (2021). Effect of rodhradi gana udavartana in the management of sthaulya (overweight) with special reference to obesity. *International Journal of Life science and Pharma Research*.

[9] Helal E. G. E., Al-Shamrani A., Abdelaziz M. A., El-Gamal M. S. (2019). Comparison between the effect of sucralose and sodium saccharin on some physiological parameters in male albino rats. *The Egyptian Journal of Hospital Medicine*.

[10] Bukhari H. M., Zahran S. E., Bakr E.-S. H., Sahibzadah F. A., Header E. A. (2021). Comparison study between drugs (orlistat and chitocal) and food supplements (green tea and apple cider vinegar) for weight loss and hepatoprotection in rats. *The Egyptian Journal of Hospital Medicine*.

[11] Rhomberg L. R., Lewandowski T. A. (2006). Methods for identifying a default cross-species scaling factor. *Human and Ecological Risk Assessment: An International Journal*.

[12] Kunst A., Draeger B., Ziegenhorn J. (1988). Colorimetric methods with glucose oxidase and peroxidase. *Methods of Enzymatic Analysis*.

[13] Matthews D. R., Hosker J. P., Rudenski A. S., Naylor B. A., Treacher D. F., Turner R. C. (1985). Homeostasis model assessment: insulin resistance and beta-cell function from fasting plasma glucose and insulin concentrations in man. *Diabetologia*.

[14] Allain C. C., Poon L. S., Chan C. S. G., Richmond W., Fu P. C. (1974). Enzymatic determination of total serum cholesterol. *Clinical Chemistry*.

[15] Fossati P., Prencipe L. (1982). Serum triglycerides determined colorimetrically with an enzyme that produces hydrogen peroxide. *Clinical Chemistry*.

[16] Demacker P. N., Vos-Janssen H. E., Hijmans A. G., van’t Laar A., Jansen A. P. (1980). Measurement of high-density lipoprotein cholesterol in serum: comparison of six isolation methods combined with enzymic cholesterol analysis. *Clinical Chemistry*.

[17] Friedewald W. T., Levy R. I., Fredrickson D. S. (1972). Estimation of the concentration of low-density lipoprotein cholesterol in plasma, without use of the preparative ultracentrifuge. *Clinical Chemistry*.

[18] Norbert W. T. (1995). *Clinical Guide to Laboratory Tests*.

[19] Tietz N. W. (1976). *Fundamentals of Clinical Chemistry*.

[20] Vermeulen A., Verdonck L., Kaufman J. M. (1999). A critical evaluation of simple methods for the estimation of free testosterone in serum. *The Journal of Clinical Endocrinology & Metabolism*.

[21] Mohamed G. A., Ibrahim S. R. M., Elkhayat E. S., El Dine R. S. (2014). Natural anti-obesity agents. *Bulletin of the Faculty of Pharmacy Cairo University*.

[22] Pal S., Khossousi A., Binns C., Dhaliwal S., Ellis V. (2011). The effect of a fibre supplement compared to a healthy diet on body composition, lipids, glucose, insulin and other metabolic syndrome risk factors in overweight and obese individuals. *British Journal of Nutrition*.

[23] Anderson J. W., Smith B. M., Gustafson N. J. (1994). Health benefits and practical aspects of high-fiber diets. *American Journal of Clinical Nutrition*.

[24] Carter J. W., Hardman W. E., Heitman D. W., Cameron I. L. (1998). Type and amount of individual dietary fibers on: serum lipid profiles, serum glucose concentration and energy intake in rats. *Nutrition Research*.

[25] Kim J. D., Han I. K., Chae B. J., Lee J. H., Park J. H., Yang C. J. (1997). Effects of dietary chromium picolinate on performance, egg, quality, serum traits and mortality rate of brown layers. *Asian-Australasian Journal of Animal Sciences*.

[26] Hashem M. A., Abd-Allah N. A., Mahmoud E. A., Amer S. A., Alkafafy M. (2022). A preliminary study on the effect of psyllium husk ethanolic extract on hyperlipidemia, hyperglycemia, and oxidative stress induced by triton X-100 injection in rats. *Biology*.

[27] Miura K., Ohnishi H., Ohira H. (2016). Innate immunity and the liver. *The Liver in Systemic Diseases*.

[28] Chaung S. S., Lin C.-C., Lin J., Yu K.-H., Hsu Y.-F., Yen M.-H. (2003). The hepatoprotective effects of *Limonium sinense* against carbon tetrachloride and beta-D-galactosamine intoxication in rats. *Phytotherapy Research*.

[29] Kumar V., Cotran R. S., Robbins S. L. (1997). *Basic Pathology*.

[30] Shafik N. M., Baalash A., Ebeid A. M. (2017). Synergistic cardioprotective effects of combined chromium picolinate and atorvastatin treatment in triton X-100-induced hyperlipidemia in rats: impact on some biochemical markers. *Biological Trace Element Research*.

[31] Fabbrini E., Sullivan S., Klein S. (2010). Obesity and nonalcoholic fatty liver disease: biochemical, metabolic, and clinical implications. *Hepatology*.

[32] El-Shetry E. S., Farag A. I. (2020). Chromium-induced hepatotoxicity and potential protective effect of selenium in adult male albino rat: a histological, immuno-histochemical and molecular study. *The Medical Journal of Cairo University*.

[33] Soudani N., Ben Amara I., Sefi M., Boudawara T., Zeghal N. (2011). Effects of selenium on chromium (VI)-induced hepatotoxicity in adult rats. *Experimental & Toxicologic Pathology*.

[34] Krzysik M., Grajeta H., Prescha A., Weber R. (2011). Effect of cellulose, pectin and chromium (III) on lipid and carbohydrate metabolism in rats. *Journal of Trace Elements in Medicine & Biology*.

[35] Ginsberg H. N. (2000). Insulin resistance and cardiovascular disease. *Journal of Clinical Investigation*.

[36] Pal S., Radavelli-Bagatini S. (2012). Effects of psyllium on metabolic syndrome risk factors. *Obesity Reviews*.

[37] Ferré P. (2004). The biology of peroxisome proliferator-activated receptors: relationship with lipid metabolism and insulin sensitivity. *Diabetes*.

[38] Loh A. H., Cohen A. H. (2009). Drug-induced kidney disease--pathology and current concepts. *Annals Academy of Medicine Singapore*.

[39] Gowda S., Desai P. B., Kulkarni SS, Hull VV, Math AA, Vernekar SN (2010). Markers of renal function tests. *North American Journal of Medical Sciences*.

[40] Baruthio F. (1992). Toxic effects of chromium and its compounds. *Biological Trace Element Research*.

[41] Warner M. H., Beckett G. J. (2010). Mechanisms behind the non-thyroidal illness syndrome: an update. *Journal of Endocrinology*.

[42] Watts D. L. (1989). The nutritional relationships of chromium. *Journal of Orthomolecular Medicine*.

